# Improving the Transferability of Adversarial Examples With a Noise Data Enhancement Framework and Random Erasing

**DOI:** 10.3389/fnbot.2021.784053

**Published:** 2021-12-09

**Authors:** Pengfei Xie, Shuhao Shi, Shuai Yang, Kai Qiao, Ningning Liang, Linyuan Wang, Jian Chen, Guoen Hu, Bin Yan

**Affiliations:** Henan Key Laboratory of Imaging and Intelligent Processing, PLA Strategy Support Force Information Engineering University, Zhengzhou, China

**Keywords:** adversarial examples, black-box attack, transfer-based attack, data enhancement, transferability

## Abstract

Deep neural networks (DNNs) are proven vulnerable to attack against adversarial examples. Black-box transfer attacks pose a massive threat to AI applications without accessing target models. At present, the most effective black-box attack methods mainly adopt data enhancement methods, such as input transformation. Previous data enhancement frameworks only work on input transformations that satisfy accuracy or loss invariance. However, it does not work for other transformations that do not meet the above conditions, such as the transformation which will lose information. To solve this problem, we propose a new noise data enhancement framework (NDEF), which only transforms adversarial perturbation to avoid the above issues effectively. In addition, we introduce random erasing under this framework to prevent the over-fitting of adversarial examples. Experimental results show that the black-box attack success rate of our method Random Erasing Iterative Fast Gradient Sign Method (REI-FGSM) is 4.2% higher than DI-FGSM in six models on average and 6.6% higher than DI-FGSM in three defense models. REI-FGSM can combine with other methods to achieve excellent performance. The attack performance of SI-FGSM can be improved by 22.9% on average when combined with REI-FGSM. Besides, our combined version with DI-TI-MI-FGSM, i.e., DI-TI-MI-REI-FGSM can achieve an average attack success rate of 97.0% against three ensemble adversarial training models, which is greater than the current gradient iterative attack method. We also introduce Gaussian blur to prove the compatibility of our framework.

## 1. Introduction

In recent years, the data-driven deep neural network (DNNs) has developed rapidly due to its excellent performance. It has made outstanding achievements in image classification (He et al., 2016; Szegedy et al., 2017), target detection (Redmon and Farhadi, 2018; Bochkovskiy et al., 2020), face recognition (Deng et al., 2019), automatic driving (Bojarski et al., 2016), natural language processing (Gehring et al., 2017; Vaswani et al., 2017) and so on. Unfortunately, the current deep learning model has been proved to be not robust, and they are vulnerable to adversarial examples. In the field of computer vision, adversarial examples are specially tailored to the target model, which can make the model misclassified but are visually similar to the original sample. Subsequently, with the development of adversarial attack and defense, its attack range is gradually expanded to speech recognition model (Carlini and Wagner, 2018), reinforcement learning model (Behzadan and Munir, 2017), graph neural network (Dai et al., 2018), etc.

The adversarial attack was first proposed by Szeged (Szegedy et al., 2013), and they use the L-BFGS optimization algorithm to find adversarial examples. Later, DeepFool (Moosavi-Dezfooli et al., 2016; Carlini and Wagner, 2017) and other optimization-based algorithms are proposed, but they focus on meeting established optimization goals in white-box attacks. However, these optimization-based methods take too much time and have poor transferability in black-box attacks. A black-box attack refers to the attack that attacker cannot know the network structure, parameters, and other information of the attacked model. Black-box attacks can be divided into three categories: scores-based, decision-based, and transfer-based attacks. In this paper, we discuss the more difficult black-box transfer attacks. Papernot et al. (2016) find that adversarial examples generated by one model can attack another model. The transferability of adversarial examples is similar to the generalization of model training. The latter is to train a robust model to classify the samples correctly, and the former is to train a robust sample so that it can successfully attack various models. Tramér et al. (2017) show that using the integrated model can train robust adversarial examples with stronger attack performance. However, simply adding pre-models requires a lot of storage space and time cost; hence researchers turn their attention to data enhancement, such as Dong et al. (2019), Lin et al. (2019), and Xie et al. (2019). These works essentially make use of the translation invariance, resize invariance, scaling invariance, and other properties of convolutional neural network (CNN), but when it exceeds a certain transformation range, the above properties will not hold, and the method based on data enhancement will fail. Based on this problem, we propose a NDEF, which solves the problem of limited change range. Specifically, we only perform input transformations against adversarial perturbations instead of the entire image. This avoids the trouble of misclassification of the original image in a wide range of changes. In addition, inspired from Zhong et al. (2020), we introduce a new data enhancement method in this framework, namely random erasing, which can effectively avoid the adversarial examples falling into an over-fitting state. Experiments show that the average success rate of our method is 4.2% higher than DI-FGSM and 2.5% higher than SI-FGSM on average, and DI-TI-MI-FGSM combined with our method can achieve an average attack success rate of 97.0% against three ensemble adversarial training models.

Our main contributions are summarized as follows.

- We propose a noise data enhancement framework (NDEF), which effectively solves the problem that some transformations, such as random erasing and Gaussian blur, that do not satisfy accuracy invariance cannot work in the previous framework. These input transformation methods can work in our framework.
- We introduce random erasing as an input transform into the gradient iterative attack for the first time and call it Random Erasing Iterative Fast Gradient Sign Method (REI-FGSM). The experimental results show that the attack success rate of our method is 4.2% higher than DI-FGSM and 2.5% higher than SI-FGSM on average. Our method can be combined with other gradient iteration methods. DI-TI-MI-REI-FGSM can achieve an average attack success rate of 97.0% against three ensemble adversarial training models, which is greater than the current gradient iterative attack method.

## 2. Related Work

### 2.1. Adversarial Attack

Szegedy et al. first produce adversarial examples using box constraint algorithm L-BFGS. However, this method requires huge costs; hence (Goodfellow et al., 2015) propose a FGSM to generate adversarial examples. This method belongs to the one-step iterative attack method, aiming to find the direction of maximizing the loss function. Subsequently, Kurakin et al. (2016) propose a multistep iterative attack method I-FGSM based on FGSM, which can ensure that the adversarial examples can find the direction of the maximum loss function in each iteration. I-FGSM can achieve excellent performance in white box attack, but the attack performance of black-box is poor. This is because I-FGSM is easy to fall into over-fitting on the substitute model. Therefore, many works begin to study how to improve the transferability of adversarial examples. At present, black-box transfer attacks can be divided into four categories, i.e., based on gradient information mining, based on data enhancement, based on model enhancement, and intermediate-layers attack.

#### 2.1.1. Gradient Information Mining Methods

Gradient information mining methods refer to various methods that attackers deal with gradient after gradient back-iteration to adjust the current gradient, propagation. Dong et al. (2018) propose MI-FGSM, which uses the momentum in the gradient iteration process to stabilize the gradient direction and escape from the local extremum. Similar to MI-FGSM, NI-FGSM (Lin et al., 2019) escapes local extremum faster by introducing Nesterov acceleration gradient. Wang and He (2021) propose variance tuning MI-FGSM, as VMI-FGSM, which uses the gradient variance of the previous iteration to adjust the current gradient, stabilize the update direction, and avoid poor local optimization in the iteration process. Wu et al. (2018) use Gaussian noise to simulate local fluctuations in substitute models to improve transferability. Gao et al. (2020) find that increasing the step size can increase the transferability, but it can lead to gradient overflow; hence, they propose PI-FGSM, which uses pre-trained convolution kernels to project the proposed overflow information to the surrounding area to improve transferability. Wu et al. (2020a) use the skip structure of the residual network to improve the transferability. Specifically, the gradient of the residual network is decomposed, and the attenuation parameter is introduced to reduce the gradient from the residual block and pay more attention to the gradient information flow from the bottom.

#### 2.1.2. Data Enhancement Methods

Data enhancement methods are methods that an attacker performs a series of transformations on a sample before entering a model to enhance transferability. DI-FGSM (Xie et al., 2019) improves the transferability of adversarial examples by introducing random resizing and random padding for input in the gradient iteration process. Using the scale invariance of CNN, SI-FGSM (Lin et al., 2019) introduces scale transformation in the gradient iteration process to improve the transferability of adversarial examples. TI-FGSM (Dong et al., 2019) uses the translation invariance of CNN and replaces the translation operation with pre-trained convolution to save substantial time and space costs. Zou et al. (2020) find that TI-FGSM can be regarded as a Gaussian blur, and the information of normal image will be lost by the Gaussian blur, while the vertical and horizontal stripes can alleviate this phenomenon. They further find that the larger the scaling ratio of DI-FGSM will generate more stripes, which will make the mitigation effect better. Based on this, they propose resized-diverse-inputs methods, which can effectively improve transferability. Wu et al. (2021) train an adversarial transformation network to replace previous transformation algorithms. Specifically, they first train an adversarial transformation network using the maximum and minimum, which can effectively correct the adversarial examples while keeping the original samples unchanged. Then they combine adversarial transformation networks with the target model and attack them. The previous work is to perturb a single image. Wang et al. (2021a) propose Admix Attack Method (AAM), which integrates some information of other categories of images into the original category to enhance transferability.

#### 2.1.3. Model Enhancement Methods

Model enhancement methods refer to the methods by which an attacker improves transferability by model integration or transformation. Liu et al. (2017) propose a model-ensemble attack method that can effectively attack robust black-box models for adversarial training. Li et al. (2020) erode the dropout layer and skip the connection layer of the model to obtain rich network models at low cost and then improve transferability through vertical integration.

#### 2.1.4. Intermediate-Layers Attack Methods

Intermediate-layers attack methods launch attacks by using information from the network middle layer instead of the logit layer. Inkawhich et al. (2020) use the Euclidean distance to reduce the discrepancy between the intermediate source and target features to achieve target attacks, but this pixel-wise Euclidean distance would impose a spatial-consistency constraint on them. To solve this problem, Gao et al. (2021) propose Pair-wise Alignment Attack (PAA) and Global-wise Alignment Attack (GAA), which use statistic alignment. Specifically, PAA uses maximum mean discrepancy (MMD) to estimate the difference between the intermediate source and target features, while GAA uses mean and variance to achieve this goal. Inkawhich et al. (2020) propose Feature Distribution Attack (FDA), which first trains a binary network to extract the feature distribution of classes and layers. Then they maximize the probability of specific classes in the auxiliary network to accomplish target attack. Wu et al. (2020b) find that the attention regions of different models are almost the same. Based on this, they propose an Attention-guided Transfer Attack (ATA) method, and add the attention region loss into the loss function to make the attention region change more to enhance transferability. Wang et al. (2021b) propose Feature Importance-aware Attack (FIA), which uses a random transformation to destroy the key features that determine the decisions of different models, and then gradient aggregation is carried out to improve transferability.

### 2.2. Adversarial Defense

Adversarial training is currently considered to be the strongest method defending adversarial examples, which add adversarial examples during model training. These works (Szegedy et al., 2013; Goodfellow et al., 2015) first mention adversarial training. Subsequently, Madry et al. (2019) analyze adversarial training from the perspective of robust optimization for the first time, propose a min-max framework, and use the adversarial examples generated by Project Gradient Descent (PGD) to achieve the approximate solution of the framework. Input transformation is another common defense method. Madry et al. (2019) find that JPEG compression can effectively suppress small perturbation adversarial examples. Xie et al. (2017) mitigates the impact of attacks by random resizing and random padding. In recent years, some works (Raghunathan et al., 2018; Fischer et al., 2020) has begun to focus on certified defense methods.

## 3. Methods

### 3.1. Problem Definition

#### 3.1.1. Adversarial Example

Suppose *x* is a clean sample, *y*^*true*^ is the corresponding real label. For a trained DNN *F*_1_, it can correctly classify samples *x* as labels *y*^*true*^. By adding a small perturbation δ to the original sample, the adversarial examples *x* + δ can make the DNN *F*_1_ misclassified. The generation of the small perturbation is generally obtained by maximizing the loss function *J*(*x, y*^*true*^, θ), where θ represents the network structure parameters, and the loss function generally selects the cross entropy loss function.

#### 3.1.2. Black-Box Transfer Attack

Assuming DNNs *F*_1_ and *F*_2_ perform the same task, which both can correctly classify clean samples *x* as labels *y*^*true*^, we denote θ_1_ θ_2_ are the network parameters of *F*_1_ and *F*_2_ respectively. In the black-box attack background, only the parameters *F*_1_ are known, and the parameters *F*_2_ are unknown. The goal of black-box attack is that the adversarial examples generated by the existing network structure information θ_1_ can make misclassification on *F*_2_, i.e., $F_{2}\left(x^{adv}\right)\neq y^{true}$.

### 3.2. Classical Attack Methods

In this section, we will briefly review the classic adversarial attack algorithms.

**Fast Gradient Sign Method:** Goodfellow et al. (2015) believe that the linear nature of the neural network leads to the generation of adversarial examples, and propose an FGSM for the first time. The purpose of this method is to find the direction of the maximum loss function. The formula is as follows :


(1) $$\begin{array}{l} x^{adv}=x+\varepsilon \cdot sign\left(\nabla_{x}L\left(x,y^{true},\theta \right)\right) \end{array}$$

**Iterative FGSM (I-FGSM):** Kurakin et al. (2016) propose an iterative version of FGSM, i.e., I-FGSM. Compared with FGSM, I-FGSM can more accurately maximize the loss function. The formula is as follows:


(2) $$\begin{array}{l} x_{0}^{{}^{adv}}=x \end{array}$$

(3) $$\begin{array}{l} x_{t+1}^{{}^{adv}}=Clip_{x}^{\varepsilon }\left\{x_{t}^{adv}+\alpha \cdot sign\left(\nabla_{x}L\left(x_{t}^{adv},y^{true},\theta \right)\right)\right\} \end{array}$$

where α represents the gradient iteration step size, and $Clip_{x}^{\varepsilon }$ means that the adversarial examples *x*^adv^ is limited to the norm ball *l*_∞_ of the original sample.

**Momentum I-FGSM (MI-FGSM):** Dong et al. (2018) introduce momentum into the gradient iteration process to stabilize the gradient update direction and escape from the local extremum. The formula is as follows:


(4) $$\begin{array}{l} g_{t+1}=\mu \cdot g_{t}+\frac{\nabla_{x}J\left(x_{t}^{adv},y^{true}\right)}{{\left||\nabla_{x}J\left(x_{t}^{adv},y^{true}\right)|\right|}_{1}} \end{array}$$

(5) $$\begin{array}{l} x_{t+1}^{adv}=Clip_{x}^{\varepsilon }\left\{x_{t}^{adv}+\alpha \cdot sign\left(g_{t+1}\right)\right\} \end{array}$$

where μ represents the attenuation factor.

**Diverse Input Iterative FGSM (DI-FGSM):** Xie et al. (2019) improve the transferability of adversarial examples by introducing input transformation. The method is as follows:


(6) $$\begin{array}{l} x_{t+1}^{adv}=Clip_{x}^{\varepsilon }\left\{x_{t}^{adv}+\alpha \cdot sign\left(\nabla_{x_{t}^{adv}}J\left(D\left(x_{t}^{adv},p\right),y^{true}\right)\right)\right\} \end{array}$$

where *D* represents the input transformation, and *p* represents the transformation probability.

**Translation-Invariant Attack Method (TI-FGSM):** Dong et al. use the translation invariance of CNN and replace translation operations with convolution kernels to improve the transferability of adversarial examples.

### 3.3. Motivation

**Figure 1:**
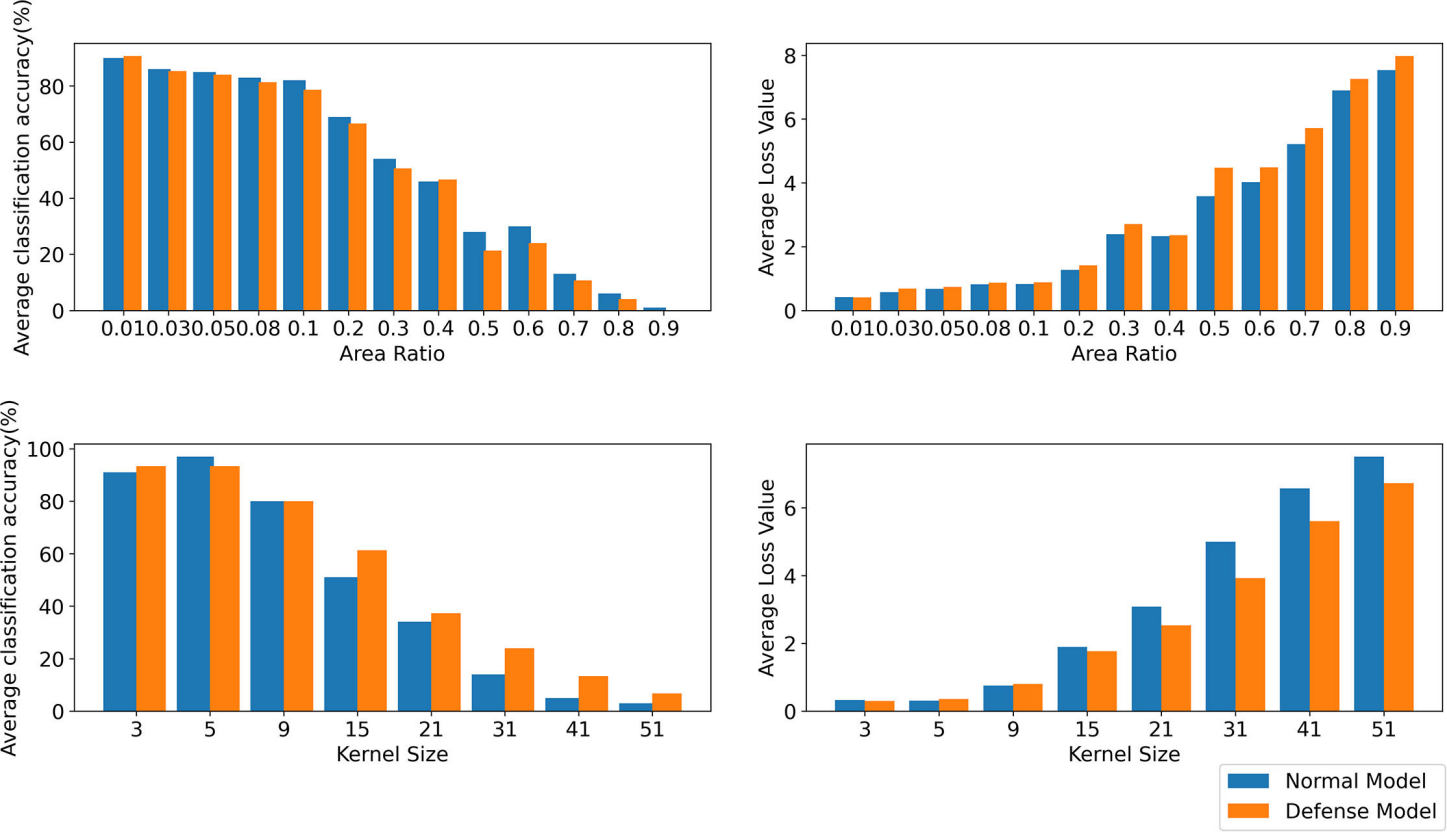
The first line shows the average classification accuracy (%) and average loss value under normal model and defense model with different area ratios by random erasing. The second line shows the average classification accuracy (%) and average loss value under normal model and defense model with different kernel sizes by Gaussian blur. The results are averaged over 1,000 images.

**Figure 2:**
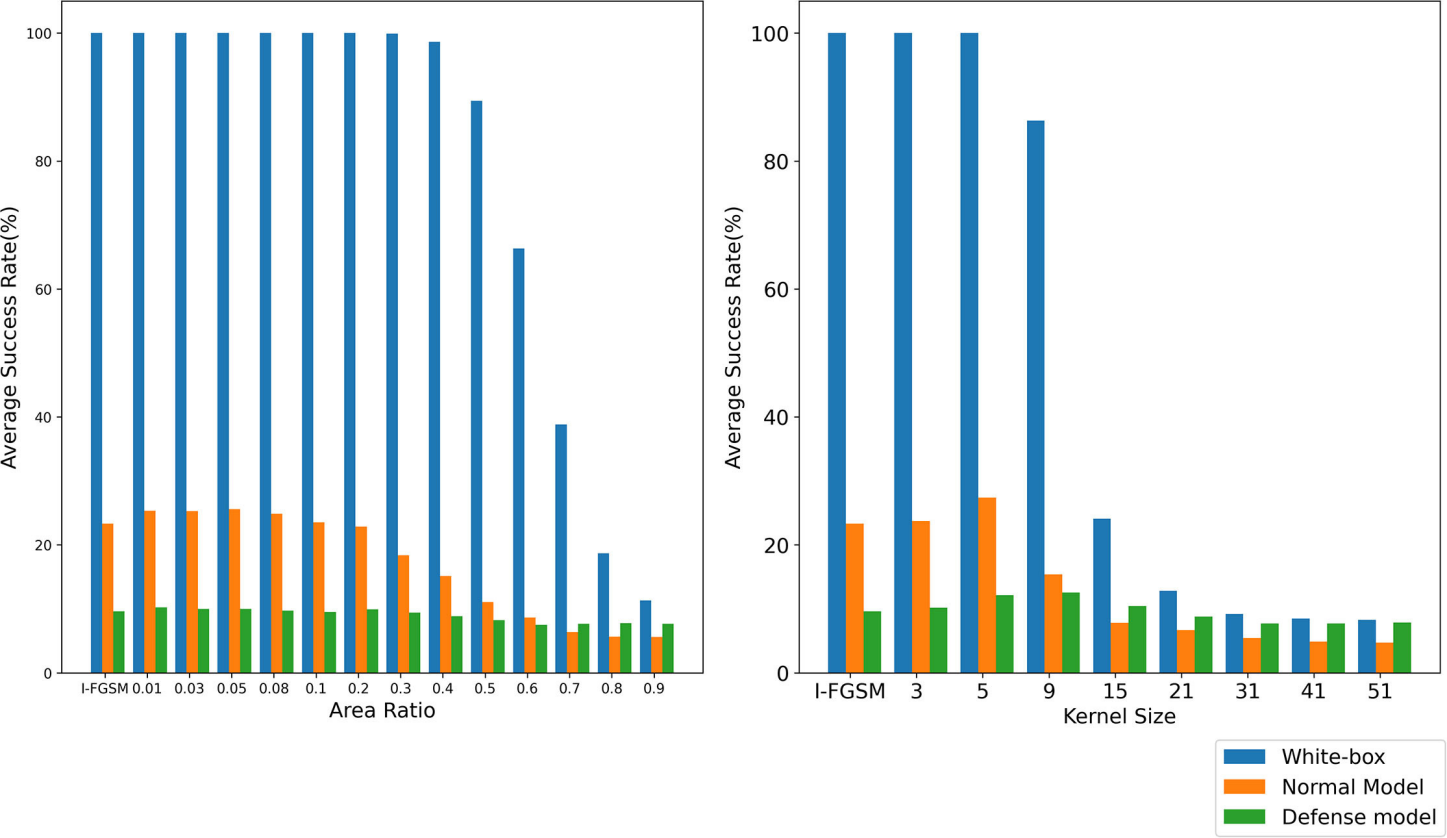
The attack success rate (%) on seven models, the adversarial examples are crafted by REI-FGSM on Inc-v3 model with different area ratios. The attack success rate (%) on seven models, the adversarial examples are crafted by random erasing and Gaussian blur on Inc-v3 model with different area ratios and kernel size in the original framework.

It is difficult to obtain good transferability by simply maximizing the loss function, such as the classical algorithm I-FGSM, because the adversarial examples generated by these methods are very easy to fall into overfitting on the substitute model in the gradient iteration process. Studies (Dong et al., 2019; Lin et al., 2019; Xie et al., 2019) have shown that the input transformation of the whole image can increase the transferability of adversarial examples. The precondition of this method is that the input transformation must satisfy certain precision invariance or loss invariance (Lin et al., 2019; Liu and Li, 2020). However, for some data enhancement methods that may lose some information, too large a transformation scale makes them unable to adapt to the above framework. We give an intuitive example by random erasing and Gaussian blur. Specifically, for random erasing, we randomly generate matrices with different area ratios from 0.01,0.03,0.05,0.08,0.1,0.2,0.3,0.4,0.5,0.6,0.7,0.8, and 0.9 and set the pixel value in the matrix to 0. For Gaussian blur, we use different kernel sizes from 3,5,9,15,21,31,41, and 51 to blur the original sample. As shown in Figure 1, the first line is the classification accuracy and loss value after random erasing, and the second line is the classification accuracy and loss value after Gaussian blur. It can be seen that when the area ratio is greater than 0.2 and the kernel size is greater than 9, the classification accuracy of CNN decreases significantly. Then, in the original framework, we test the attack success rate of random erasing and Gaussian blur under different transformation scales. As shown in Figure 2, the experimental results show that when the area ratio is greater than 0.05, the black-box attack success rate decreases. When the area rate is greater than 0.4, the black-box attack success rate decreases significantly. For Gaussian blur, when the Gaussian kernel is greater than 9, the black box attack rate decreases cliff-like. The experimental results show that the previous framework does not apply to some data enhancement methods with too large transformation scale. Based on this problem, we propose a noise data enhancement framework. Since our framework only transforms against perturbation, the structure information of the original sample will not be destroyed, which can maintain the accuracy invariance. In addition, the transformation of adversarial perturbation can hinder the generation of adversarial examples and prevent over-fitting. Our framework is a supplement to the previous framework, which can mine the potential of some transformation methods without accuracy invariance in transfer attack methods. In this paper, we mainly introduce random erasing. As far as we know, it is the first time that random erasing has been introduced into a transfer attack as an input transformation. Random erasing is an effective data enhancement method. Specifically, the rectangular region of the image is randomly selected, and the pixels are erased or replaced by other values. The generation of adversarial examples with occlusion levels will reduce the risk of overfitting and make the adversarial examples robust to occlusion. In addition, in order to verify that our framework can also be compatible with other methods, we briefly introduce Gaussian blur.

**Algorithm 1:** REI-FGSM

	**Input**	: An original image *x*, normalized to [−1, 1] and corresponding true labels *y*^*true*^; maximum perturbation value ε; iteration rounds *T*; adversarial perturbation δ_*t*_, input image size *W*, *H*; lower bound θ_*L*_, upper bound θ__*H*__ of mask matrix area ratio; number of matrices *K*.
	**Output**	: An adversarial example *x*_*adv*_.
1	$a=\frac{\varepsilon }{T}$;	
2	Initialize $x_{0}^{adv}=x$;	
3	Random initialization adversarial perturbation δ_0_;	
4	**for** *t* ← 0 **to** *T* − 1 **do**	
5	Replicate adversarial perturbation δ_*t*_ and get adversarial perturbation${\delta_{\text{t}}}^{*}$;	
6	Get the area ratio of random masking matrix θ_*e*_ = *Rand*(θ_*L*_, θ_*H*_);	
7	Get the area of random masking matrix *S*_*e*_ = W*H*θ_*e*_;	
8	**for** *i* ← 0 **to** *K* − 1 **do**	
9	if *random*(1)>0.5 then	
10	Get the aspect ratio of the *jth* matrix	
	φ__*e*__ = *Rand*(θ_*e*_, 1);	
11	else:	
12	Get the aspect ratio of the *jth* matrix $\varphi_{e}=Rand\left(1,\frac{1}{\theta_{e}}\right)$;	
13	Get the *jth* matrix length $H_{j}=Floor\left(\sqrt{\frac{S_{e}}{\varphi_{j}}}\right)$;	
14	Get the *jth* matrix width $W_{j}=Floor\left(\sqrt{S_{e}*\varphi_{j}}\right)$;	
15	Get the horizontal ordinate of starting pixels of *jth* matrix *X*_*j*_ = *Rand*(0, (*H* − *H*_*j*_));	
16	Get the ordinate of starting pixels of *jth* matrix *Y*_*j*_ = *Rand*(0, (*W* − *W*_*j*_));	
17	Set 0 for region [*X*_*j*_ + *H*_*j*_, *Y*_*j*_ + *W*_*j*_] in ${\delta_{\text{t}}}^{*}$;	
18	**end**	
19	Calculate gradient $\nabla_{\delta_{t}}J\left(\left(x+\delta_{t}^{*}\right),y^{true}\right)$;	
20	Update adversarial perturbation $\delta_{t}\text{=}\delta_{t}\text{+}\alpha \cdot \text{sign(}\nabla_{\delta_{t}}J\left(\left(x+\delta_{t}^{*}\right),y^{true}\right)\text{)}$;	
21	Clip the adversarial perturbation δ_*t*_ = *Clip*(δ_*t*_, −ε, ε);	
22	Get adversarial examples $x_{t}^{adv}=x+\delta_{t}$;	
23	Clip the adversarial examples $x_{t}^{adv}=Clip\left(x_{t}^{adv},-1,1\right)$;	
24	Get adversarial perturbation $\delta_{t}=x_{t}^{adv}-x$;	
25	**end**	
26	Return $x_{t}^{adv}=x+\delta_{t}$;	

**Algorithm 2:** GBI-FGSM

	**Input**	: An original image *x*, normalized to [−1, 1] and corresponding true labels *y*^*true*^; maximum perturbation value ε; iteration rounds *T*; adversarial perturbation δ_*t*_; the kernel size *k*; Output: An adversarial example *x*_*adv*_.
	**Output**	: An adversarial example *x*_*adv*_.
1	$a=\frac{\varepsilon }{T}$;	
2	Initialize $x_{0}^{adv}=x$;	
3	Random initialization adversarial perturbation δ_0_;	
4	**for** *t* ← 0 **to** *T* − 1 **do**	
5	Replicate adversarial perturbation δ_*t*_ and get adversarial perturbation ${\delta_{\text{t}}}^{*}$;	
6	Gaussian blur for adversarial perturbation and update $\delta_{t}^{*}=Gaussianblur\left(\delta_{t}^{*},k\right)$;	
7	Calculate gradient $\nabla_{\delta_{t}}J\left(\left(x+\delta_{t}^{*}\right),y^{true}\right)$;	
8	Update adversarial perturbation $\delta_{t}\text{=}\delta_{t}\text{+}\alpha \cdot \text{sign(}\nabla_{\delta_{t}}J\left(\left(x+\delta_{t}^{*}\right),y^{true}\right)\text{)}$;	
9	Clip the adversarial perturbation δ_*t*_ = *Clip*(δ_*t*_, −ε, ε);	
10	Get adversarial examples $x_{t}^{adv}=x+\delta_{t}$;	
11	Clip the adversarial examples $x_{t}^{adv}=Clip\left(x_{t}^{adv},-1,1\right)$;	
12	Get adversarial perturbation $\delta_{t}=x_{t}^{adv}-x$;	
13	**end**	
14	Return $x_{t}^{adv}=x+\delta_{t}$;	

### 3.4. Framework

As far as we know, the current data-enhanced attack methods generally have to satisfy the invariance property as follows:


(7) $$\begin{array}{l} \arg\max(\left(F_{Logit}\left(x\right)\right)=\arg\max\left(F_{Logit}\left(T\left(x\right)\right)\right) \end{array}$$

Meanwhile, input transformation destroys the structure of the adversarial example to remove or weaken its attack performance, which can effectively enhance the diversity of model output. This can be described as the following formula:


(8) $$\begin{array}{l} F_{Logit}\left(x^{adv}\right)\neq F_{Logit}\left(T\left(x^{adv}\right)\right) \end{array}$$

where *T*(·) represents a certain transformation and *F*_*Logit*_ represents the logit output of the model. Lin et al. (2019) and Liu and Li (2020) interpret that model augmentation can be achieved by loss-preserving transformation and accuracy-maintained transformation. However, some transformations that do not meet the CNN invariant characteristics will fail in this framework. In order to make these transformations also play their performance, in this paper, we propose a new data enhancement framework, only aimed at adversarial perturbation, and we replace *F*_*Logit*_(*T*(*x* + δ)) with *F*_*Logit*_(*x* + *T*(δ)), so that the original sample will not be disturbed.

Meanwhile, the input transformation will affect the adversarial perturbation, thus affecting the logit output of the model. The formula is shown below.


(9) $$\begin{array}{l} F_{Logit}\left(x+T\left(\delta \right)\right)\neq F_{Logit}\left(x+\delta \right) \end{array}$$

We use *M* to represent the model space for the same task; *F* is a model in this space. Since the adversarial perturbation is interfered by the input transformation, the logit output of *F* changes. We can find another model F^*^ in this space to make its logit output approximate to the logit output of *F*. The formula is shown below.


(10) $$\begin{array}{l} F_{Logit}^{*}\left(x+\delta \right)\approx F_{Logit}\left(x+T\left(\delta \right)\right) \end{array}$$

**Figure 3:**
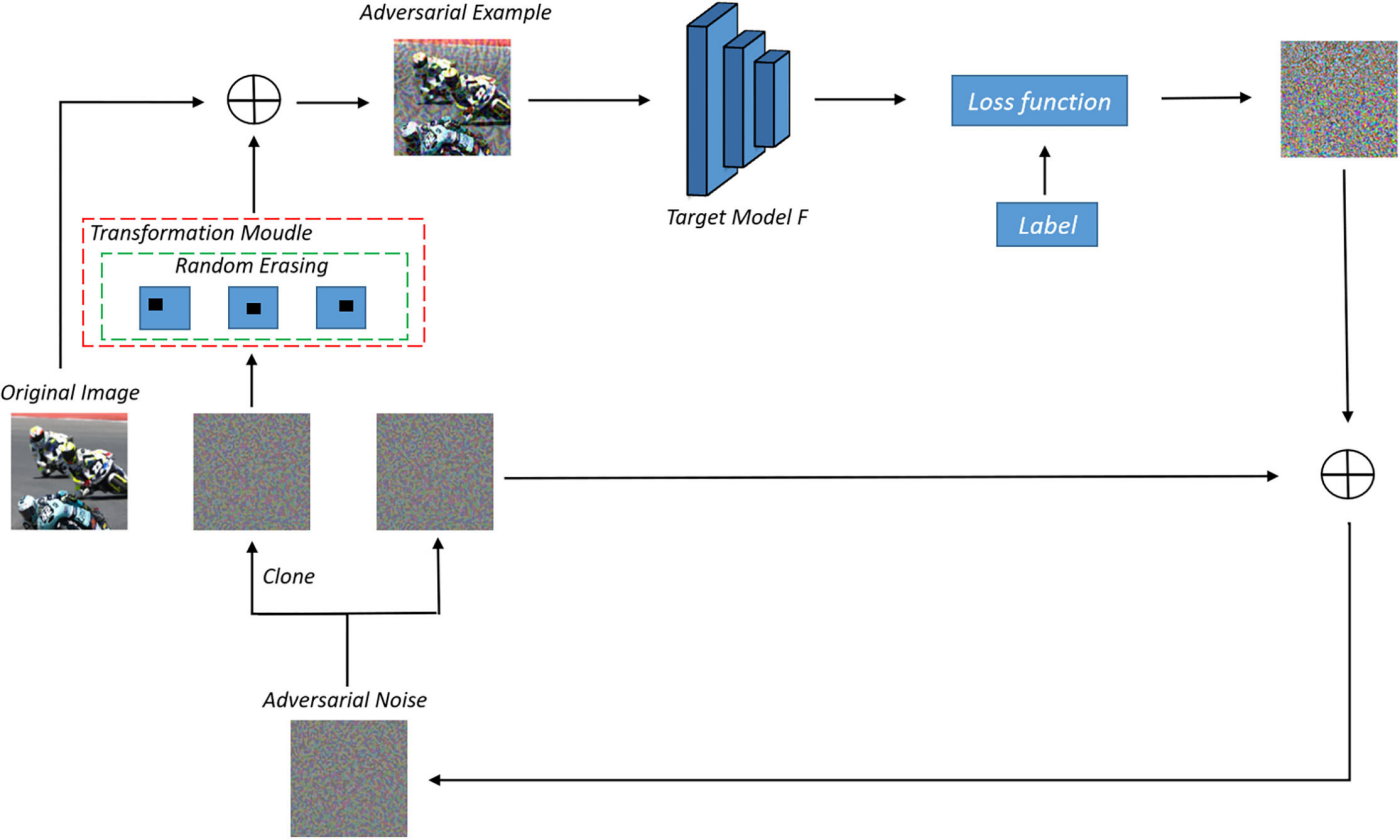
The framework of our methods.

In other words, we use the above framework to change the logit output of the substitute model during each iteration to achieve model augmentation. Our frame diagram is shown in Figure 3. Specifically, we copy the adversarial perturbation, one for storing the previous adversarial perturbation information, and one for data enhancement. Here, we introduce random erasing. We study single matrix erasing and multi-matrix erasing, respectively. Specifically, we select randomly the area ratio within a finite interval in each iteration, then select randomly the aspect ratio within the interval confirmed by the area ratio, finally, initialize the starting point of the matrix randomly. The pixels of the matrix can be set to 0, or other values. In this paper, we set the pixel of the erased matrix to 0. The specific algorithm is shown in Algorithm 1. In addition, our framework can also be combined with previous methods for the whole image enhancement.

To further verify that our framework can be combined with other algorithms, we introduce Gaussian blur (Gedraite and Hadad, 2011) and call it the Gaussian Blur Iterative FGSM (GBI-FGSM). We prove that using Gaussian blur on the previous framework is not very good, while Gaussian blur in our framework can get relatively good performance, especially on defense models. This is because Gaussian blur in the original framework will lose a large number of original sample information, but our framework can effectively prevent this. We call the operation of Gaussian blur *Gaussianblur*(·). Our algorithm is shown in Algorithm 2.

## 4. Experiment

**Dataset:** Following previous works (Dong et al., 2018; Lin et al., 2019; Xie et al., 2019), we select the NIPS2017 competition dataset. This dataset extracted 1,000 natural images from the ImageNet dataset and adjusted their size to 299 × 299 × 3.

**Network:** We selected seven models as our experimental models, including four models under natural training, i.e., Inception-v3 (Inc-v3) (Szegedy et al., 2016), Inception-v4 (Inc-v4) InceptionResnet-v2 (IncRes-v2) (Szegedy et al., 2017), Resnet-v2- 152 (Res-152) (He et al., 2016), and three ensemble adversarial training model (Tramér et al., 2017), i.e., ens3-adv-Inception-v3 (Inc-v3ens3), ens4-adv-Inception-v3 (Inc-v3ens4), and ens-adv-Inception-ResNet-v2 (IncRes-v2ens).

**Experimental details:** In our experiment, we compare I-FGSM, DI-FGSM, MI-FGSM, SI-FGSM, TI-FGSM, PI-FGSM, and their combined versions, i.e., DI-TI-MI-FGSM, REI-TI-MI-FGSM, and DI-TI-MI-REI-FGSM in the scenario of non-targeted attacks. In our experiment, we set the number of gradient iterations *T* to 10, the step size α to 1.6, and max perturbation ε to 16. For MI-FGSM, we set the delay factor μ = 1.0; for TI-BIM, we set the kernel size *k* = 15; for DI-FGSM, we set the conversion probability *p* = 0.7; for SI-FGSM, the number of the scale copies *m* is set to 5; and for PI-FGSM, we set the amplification factor β = 10.

### 4.1. The Number and Area of Erasing Matrix

In this section, we discuss the attack performance of the number and area of erasing matrices. Specifically, we choose Inc-v3 as a substitute model to generate adversarial examples and test the results under the other six models with the variable-controlled methods. According to the work by Xie et al. (2021), we set *T* = 50, *a* = 1.6, and ε = 16.

#### 4.1.1. Area of Erasing Matrix

**Figure 4:**
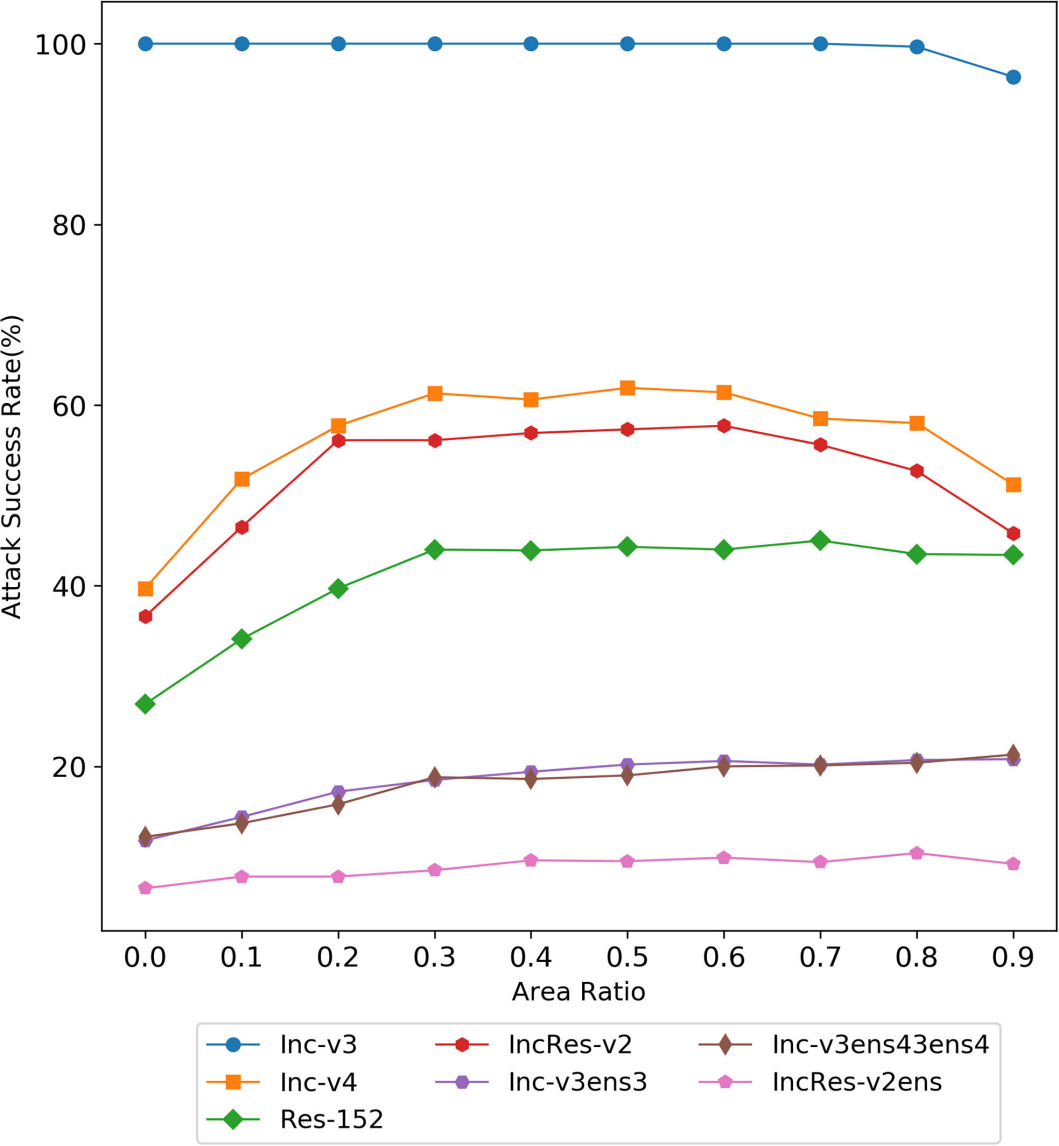
The attack success rate (%) on seven models, the adversarial examples are crafted by Random Erasing Iterative Fast Gradient Sign Method (REI-FGSM) on Inc-v3 model with different area ratios.

Here, we discuss the attack performance under the erasing of a single matrix with different erasing area ratios. As shown in Figure 4, with the increase of erasing area, the black-box attack success rate of the three normal models first increases and then remains basically unchanged or slightly decreases, while the attack success rate of the three defense models basically continues to rise. When the erasure area ratio is 0.9, our method can still maintain a high attack success rate, while the attack success rate of the previous framework will decrease very low, indicating the effectiveness of our method. In the normal training model, the attack performance is the best when the erasing area ratio is of 0.5, and in the ensemble adversarial training model, the attack performance is the best when the erasing area ratio is 0.8.

#### 4.1.2. Numer of Erasing Matrix

**Figure 5:**
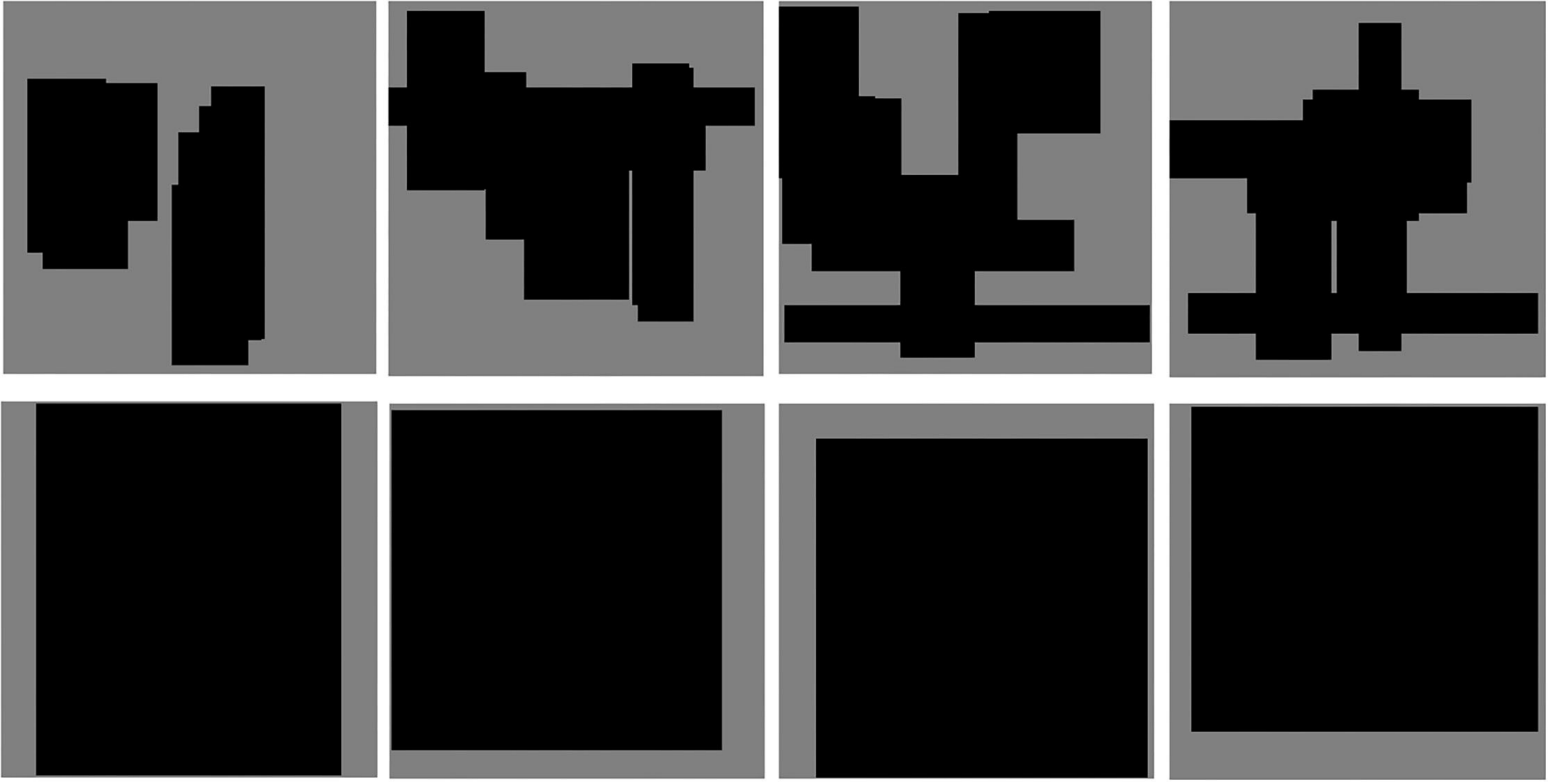
Comparison of multi-matrix erasing (top) and single-matrix erasing (bottom).

In this subsection, we discuss the attack performance under different number of erasing matrices with erasing area ratio 0.1. As shown in Table 1, with the increase of the number of matrices, the success rate of black-box attack begins to increase. When the number of matrices is 8, the attack on the normal model is the best, and when the number of matrices is 15, the attack on the ensemble adversarial training model is the best. Even if the total erasing area ratio has exceeded 1.0, it can still maintain a high attack success rate, because the initial point of the matrix is randomly selected, and some matrices will overlap so that it does not cover all regions. As shown in Figure 5, multiple matrices erasing can transform more shapes than single matrix erasing. We find that when the total area is certain, using more small matrices can achieve better attack results. When the total matrix area is 0.8, the attack success rate of multi-matrix is 2.3% higher than that of a single matrix, and the best attack of multi-matrix is 4.8% higher than that of a single matrix.

**Table 1:** The attack success rate (%) of seven models, the leftmost column represents the number of erased matrices whose erased area ratio is 0.1, adversarial examples crafted by REI-FGSM on Inc-v3 model (“*” indicate the white box attack).

**Area_number**	**Inc-v3**	**Inc-v4**	**Res-152**	**IncRes-v2**	**Inc-v3ens3**	**Inc-v3ens4**	**IncRes-v2ens**
1	100.0*	51.8	34.1	46.5	14.4	13.7	7.8
3	100.0*	62.2	43.7	56.3	17.2	17.2	9.0
5	100.0*	67.2	48.9	60.7	22.2	17.5	9.6
8	100.0*	**69.2**	51.7	65.4	23.0	21.1	10.8
10	100.0*	67.9	**52.3**	**65.7**	22.5	21.5	10.3
15	100.0*	66.4	50.3	62.7	**23.9**	**22.5**	**10.9**
20	99.9*	64.5	48.6	59.8	21.9	21.9	10.8

*The bold value represents the highest success rate for different attack methods under the same experimental conditions*.

### 4.2. Attack Single Model

In this section, we compare our algorithm with the I-FGSM and data enhancement methods, such as DI-FGSM, SI-FGSM. We also test the experimental results of REI-FGSM combined with MI-FGSM, PI-FGSM and SI-FGSM. The experimental parameters follow the original paper. For REI-FGSM, we set the θ_*L*_ = θ_*H*_ = 0.1 and the number of matrices *K* = 8. When combining with PI-FGSM and SI-FGSM, we set θ_*L*_ = θ_*H*_ = 0.3 and *K* = 3 for REI-FGSM. When combining with MI-FGSM, we set θ_*L*_ = θ_*H*_ = 0.1 and *K* = 8 for REI-FGSM. As shown in Table 2, the experimental results show that the attack success rate of our method is 17.3% higher than the I-FGSM on average, 4.2% higher than the DI-FGSM and 2.5% than SI-FGSM. In the defense model, our method is 6.6% higher than DI-FGSM. As shown in Table 3, the attack performance of MI-FGSM can be improved by 5.2% on average when combined with REI-FGSM, the attack performance of SI-FGSM can be improved by 22.9% on average when combined with REI-FGSM, and the attack performance of PI-FGSM can be improved by 4.0% on average when combined with REI-FGSM. To sum up, we can find that our method can combine with the above classical methods to achieve greater performance, especially with SI-FGSM, which can increase by an average of 22.9%.

**Table 2:** The success rate(%) of non-targeted attacks of seven models.

**Model**	**Attacks**	**Inc-v3**	**Inc-v4**	**Res-152**	**IncRes-v2**	**Inc-v3ens3**	**Inc-v3ens4**	**IncRes-v2ens**
Inc-v3	I-FGSM	100.0*	29.6	19.4	20.3	11.7	12.1	5.5
	DI-FGSM	99.8*	54.2	32.1	43.6	15.0	16.2	7.1
	SI-FGSM	100.0*	50.5	38.0	44.9	21.6	**21.7**	10.0
	REI-FGSM	99.7*	**56.5**	**39.6**	**48.8**	**23.8**	21.4	**11.3**
Inc-v4	I-FGSM	43.3	100.0*	25.5	25.3	11.8	13.0	6.6
	DI-FGSM	66.6	100.0*	39.8	50.4	14.7	17.7	8.4
	SI-FGSM	69.9	100.0*	**48.1**	55.3	**26.9**	**26.5**	**14.9**
	REI-FGSM	**72.1**	99.8*	46.7	**56.2**	23.8	23.5	14.0
Res-152	I-FGSM	30.7	24.7	99.5*	16.9	13.0	13.3	6.7
	DI-FGSM	**60.0**	**56.5**	99.2*	**49.3**	21.6	21.1	12.9
	SI-FGSM	43.0	36.3	99.7*	30.6	20.5	19.2	11.6
	REI-FGSM	49.7	45.2	99.0*	40.1	**25.9**	**25.0**	**16.3**
IncRes-v2				I-FGSM	48.2	38.3	25.5	100.0*	13.7	13.3	8.2
DI-FGSM	70.2	66.1	47.9	99.2*	19.3	20.2	12.7
SI-FGSM	71.5	58.4	49.8	100.0*	**30.6**	**28.8**	**22.5**
REI-FGSM	**72.9**	**66.8**	**51.1**	99.2*	30.3	28.3	**22.5**

The top row models are substitute models, and we use them to generate adversarial examples by I-FGSM, DI-FGSM, SI-FGSM, and REI-FGSM (“*” indicates the white-box attack). The bold value represents the highest success rate for different attack methods under the same experimental conditions.

**Table 3:** The success rate(%) of non-targeted attacks of seven models.

**Model**	**Attacks**	**Inc-v3**	**Inc-v4**	**Res-152**	**IncRes-v2**	**Inc-v3ens3**	**Inc-v3ens4**	**IncRes-v2ens**
Inc-v3	MI-FGSM	100.0*	55.5	45.3	51.8	22.4	21.0	10.8
	MI-REI-FSGM	99.9	**64.1**	**51.9**	**60.5**	**26.0**	**24.7**	**13.0**
	PI-FGSM	100.0*	58.6	46.9	50.3	31.4	31.8	20.1
	PI-REI-FGSM	100.0*	**64.4**	**51.5**	**57.5**	**34.3**	**32.4**	**21.7**
	SI-FGSM	100.0*	50.5	38.0	44.9	21.6	**21.7**	10.0
	SI-REI-FGSM	99.4*	**78.0**	**65.0**	**74.8**	**44.8**	**45.1**	**26.4**
Inc-v4	MI-FGSM	71.0	100.0*	51.5	58.4	24.1	23.1	14.0
	MI-REI-FSGM	**78.0**	100.0*	**57.7**	**65.2**	**28.8**	**27.6**	**16.9**
	PI-FGSM	71.6	100.0*	50.2	54.4	35.4	35.2	25.0
	PI-REI-FGSM	**76.0**	99.9*	**54.9**	**63.4**	**37.3**	**37.9**	**26.3**
	SI-FGSM	69.9	100.0*	48.1	55.3	26.9	26.5	14.9
	SI-REI-FGSM	**86.6**	98.9*	**73.2**	**78.5**	**54.0**	**50.5**	**36.1**
Res-152	MI-FGSM	57.5	51.2	99.2*	47.0	27.1	24.8	15.6
	MI-REI-FSGM	**60.3**	**55.9**	99.2*	**52.6**	**30.9**	**30.0**	**18.8**
	PI-FGSM	63.6	54.5	99.7*	50.8	37.5	36.9	26.7
	PI-REI-FGSM	**66.1**	**59.4**	99.3*	**54.8**	**41.0**	**40.4**	**29.4**
	SI-FGSM	43.0	36.3	99.7*	30.6	20.5	19.2	11.6
	SI-REI-FGSM	**61.8**	**58.1**	97.9*	**54.4**	**40.5**	**38.1**	**27.8**
IncRes-v2	MI-FGSM	77.7	67.0	58	100.0*	31.6	28.1	20.7
	MI-REI-FSGM	**81.6**	**74.9**	**64.3**	99.7*	**38.4**	**33.9**	**24.3**
	PI-FGSM	76.3	69.4	59.0	100.0*	40.8	39.1	32.0
	PI-REI-FGSM	**80.6**	**73.9**	**66.1**	99.8*	**45.4**	**43.5**	**36.1**
	SI-FGSM	71.5	58.4	49.8	100.0*	30.6	28.8	22.5
	SI-REI-FGSM	**84.8**	**80.7**	**76.3**	98.6*	**61.5**	**54.9**	**48.2**

The top row models are substitute models, and we use them to generate adversarial examples by MI-FGSM, PI-FGSM, SI-FGSM, and thier combination with REI-FGSM, (“*” indicates the white box attack). The bold value represents the highest success rate for different attack methods under the same experimental conditions.

### 4.3. Attack Ensemble Model

In this section, we use DI-TI-MI-FGSM, REI-TI-MI-FGSM, and DI-TI-MI-REI-FGSM to attack four normal models, and test the success rate of the black-box attack on three ensemble adversarial training models. Following the work (Xie et al., 2021), we set *T* = 50, *a* = 3.2 and ε = 16. For REI-FGSM, we set the θ_*L*_ = θ_*H*_ = 0.01 and the number of matrices *K* = 30. As shown in Table 4, REI-TI-MI-FGSM achieves an average attack success rate of 93.1% on three defense models, which is 0.5% higher than DI-TI-MI-FGSM. The average attack performance of DI-TI-MI-REI-FGSM can reach 97.0%, which is 4.4% higher than that of DI-TI-MI-FGSM. As far as we know, DI-TI-MI-REI-FGSM achieves the best performance of the current attack method based on gradient iteration.

**Table 4:** The success rate(%) of non-targeted attacks of three ensemble adversarial training models.

**Model**	**Attacks**	**Inc-v3ens3**	**Inc-v3ens4**	**IncRes-v2ens**
Ensemble	DI-TI-MI-FGSM	94.8	94.5	88.5
REI-TI-MI-FGSM	94.8	94.5	89.9
DI-TI-MI-REI-FGSM	**97.6**	**97.3**	**96.2**

*The adversarial examples are crafted by DI-TI-MI-FGSM, REI-TI-MI-FGSM, and DI-TI-MI-REI-FGSM on four normal models. The bold value represents the highest success rate for different attack methods under the same experimental conditions*.

### 4.4. Compatibility of the Attack Framework

**Figure 6:**
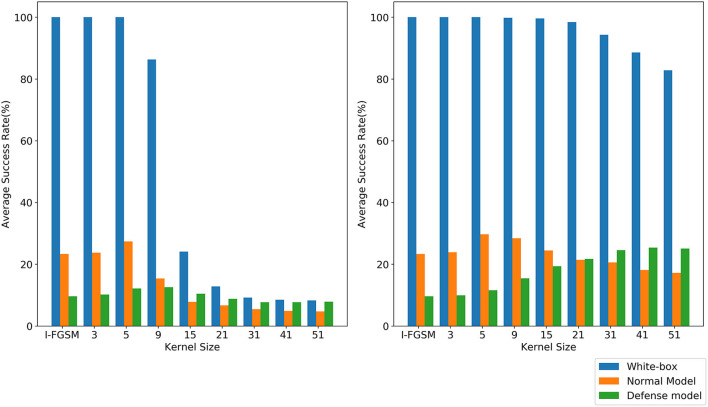
Comparison of GBI-FGSM-F (left) and GBI-FGSM (right).

In order to verify the compatibility of our framework, Gaussian blur (Gedraite and Hadad, 2011) is introduced into our framework. We make use of Gaussian blur attack inc-v3 model in the original framework and our framework, respectively, called GBI-FGSM-F and GBI-FGSM. We take the kernel size as 3,5,9,15,21,31,41, and 51 and compare it with the baseline I-FGSM. As shown in Figure 6, with the increase of kernel size, the attack success rate of GBI-FGSM-F decreases significantly, but GBI-FGSM can still maintain a high attack success rate. Although the attack success rate of GBI-FGSM on the normal model will decrease, the attack success rate on the ensemble adversarial training will increase. We believe that a large degree of disruption for adversarial perturbation during the gradient iteration may result in more robust adversarial examples against defense models. When the kernel size is 51, the attack success rate of GBI-FGSM on the three defense models can reach an average of 25.0%.

## 5. Conclusion

Previous data enhancement frameworks only work on input transformations that satisfy accuracy or loss invariance. However, it does not work for other transformations that do not meet the above conditions, such as the transformation which will lose information. In this paper, we propose a data enhancement framework only for adversarial perturbation, which can effectively solve the above problems. In addition, we introduce random erasing as an input transformation into the generation of adversarial examples for the first time. Compared with the methods based on data enhancement, such as DI-FGSM and SI-FGSM, the attack success rate of REI-FGSM can be improved by 4.2% and 2.5% on average, respectively. DI-TI-MI-REI-FGSM can achieve an average attack success rate of 97.0% on the ensemble adversarial training models, which is better than the current gradient-based iterative method. In addition, we also briefly introduce Gaussian blur to illustrate the compatibility of our framework.

## Data Availability Statement

Publicly available datasets were analyzed in this study. This data can be found here: Kaggle, https://www.kaggle.com/c/nips-2017-non-targeted-adversarial-attack/data.

## Author Contributions

All authors listed have made a substantial, direct, and intellectual contribution to the work and approved it for publication.

## Funding

This work was supported by the National Key R&D Program of China under Grant 2017YFB1002502 and National Natural Science Foundation of China (Nos. 61701089, 61601518, and 61372172).

## Conflict of Interest

The authors declare that the research was conducted in the absence of any commercial or financial relationships that could be construed as a potential conflict of interest.

## Publisher's Note

All claims expressed in this article are solely those of the authors and do not necessarily represent those of their affiliated organizations, or those of the publisher, the editors and the reviewers. Any product that may be evaluated in this article, or claim that may be made by its manufacturer, is not guaranteed or endorsed by the publisher.

## References

[B1] Behzadan V. Munir A. (2017). Vulnerability of deep reinforcement learning to policy induction attacks, in International Conference on Machine Learning and Data Mining in Pattern Recognition (Cham: Springer), 262–275.

[B2] Bochkovskiy A. Wang C.-Y. Liao H.-Y. M. (2020). Yolov4: Optimal speed and accuracy of object detection. arXiv [Preprint] arXiv:2004.10934.34300543

[B3] Bojarski M. Del Testa D. Dworakowski D. Firner B. Flepp B. Goyal P.. (2016). End to end learning for self-driving cars. arXiv [Preprint] arXiv:1604.07316.30325645

[B4] Carlini N. Wagner D. (2017). Towards evaluating the robustness of neural networks. arXiv [Preprint] arXiv: 1608.04644. 10.1109/SP.2017.4927295638

[B5] Carlini N. Wagner D. (2018). Audio adversarial examples: Targeted attacks on speech-to-text, in 2018 IEEE Security and Privacy Workshops (SPW) (San Francisco, CA: IEEE), 1–7.

[B6] Dai H. Li H. Tian T. Huang X. Wang L. Zhu J.. (2018). Adversarial attack on graph structured data, in International Conference on Machine Learning (Stockholm: PMLR), 1115–1124.

[B7] Deng J. Guo J. Xue N. Zafeiriou S. (2019). Arcface: Additive angular margin loss for deep face recognition, in Proceedings of the IEEE/CVF Conference on Computer Vision and Pattern Recognition (Long Beach, CA: IEEE), 4690–4699.

[B8] Dong Y. Liao F. Pang T. Su H. Zhu J. Hu X.. (2018). Boosting adversarial attacks with momentum, in Proceedings of the IEEE Conference on Computer Vision and Pattern Recognition (Salt Lake City, UT: IEEE), 9185–9193.

[B9] Dong Y. Pang T. Su H. Zhu J. (2019). Evading defenses to transferable adversarial examples by translation-invariant attacks, in Proceedings of the IEEE/CVF Conference on Computer Vision and Pattern Recognition (IEEE), 4312–4321.

[B10] Fischer M. Baader M. Vechev M. (2020). Certified defense to image transformations via randomized smoothing. arXiv [Preprint] arXiv:2002.12463.

[B11] Gao L. Cheng Y. Zhang Q. Xu X. Song J. (2021). Feature space targeted attacks by statistic alignment, in Proceedings of the Thirtieth International Joint Conference on Artificial Intelligence (Montreal, Canada. International Joint Conferences on Artificial Intelligence Organization), 671–677.

[B12] Gao L. Zhang Q. Song J. Liu X. Shen H. T. (2020). Patch-wise attack for fooling deep neural network, in European Conference on Computer Vision (Cham: Springer), 307–322.

[B13] Gedraite E. S. Hadad M. (2011). Investigation on the effect of a gaussian blur in image filtering and segmentation. In Proceedings ELMAR-2011, pages 393-396. IEEE.

[B14] Gehring J. Auli M. Grangier D. Yarats D. Dauphin Y. N. (2017). Convolutional sequence to sequence learning, in International Conference on Machine Learning (PMLR), 1243–1252.

[B15] Goodfellow I. J. Shlens J. Szegedy C. (2015). Explaining and harnessing adversarial examples. arXiv [Preprint] arXiv: 1412.6572.

[B16] He K. Zhang X. Ren S. Sun J. (2016). Deep residual learning for image recognition, in Proceedings of the IEEE Conference on Computer Vision and Pattern Recognition (Las Vegas, NV: IEEE), 770–778.

[B17] Inkawhich N. Liang K. J. Carin L. Chen Y. (2020). Transferable perturbations of deep feature distributions. arXiv [preprint] arXiv:2004.12519

[B18] Kurakin A. Goodfellow I. Bengio S. (2016). Adversarial examples in the physical world. arXiv [preprint] arXiv:1607.02533

[B19] Li Y. Bai S. Zhou Y. Xie C. Zhang Z. Yuille A. (2020). Learning transferable adversarial examples via ghost networks, in Proceedings of the AAAI Conference on Artificial Intelligence, Vol. 34, 11458–11465.

[B20] Lin J. Song C. He K. Wang L. Hopcroft J. E. (2019). Nesterov accelerated gradient and scale invariance for adversarial attacks. arXiv [Preprint] arXiv:1908.06281.

[B21] Liu W. Li Z. (2020). Enhancing adversarial examples with flip-invariance and brightness-invariance, in International Conference on Security and Privacy in Digital Economy (Quzhou: Springer), 469–481.

[B22] Liu Y. Chen X. Liu C. Song D. (2017). Delving into transferable adversarial examples and black-box attacks. arXiv[ Preprint] arXiv: 1611.02770.

[B23] Madry A. Makelov A. Schmidt L. Tsipras D. Vladu A. (2019). Towards deep learning models resistant to adversarial attacks. arXiv [Preprint] arXiv: 1706.06083.

[B24] Moosavi-Dezfooli S.-M. Fawzi A. Frossard P. (2016). Deepfool: a simple and accurate method to fool deep neural networks, in Proceedings of the IEEE Conference on Computer Vision and Pattern Recognition (Las Vegas, NV: IEEE), 2574–2582.

[B25] Papernot N. McDaniel P. Goodfellow I. (2016). Transferability in machine learning: from phenomena to black-box attacks using adversarial samples. arXiv [Preprint] arXiv:1605.07277.

[B26] Raghunathan A. Steinhardt J. Liang P. (2018). Certified defenses against adversarial examples. arXiv [Preprint] arXiv:1801.09344.

[B27] Redmon J. Farhadi A. (2018). Yolov3: An incremental improvement. arXiv arXiv [Preprint] arXiv:1801.09344.

[B28] Szegedy C. Ioffe S. Vanhoucke V. Alemi A. (2017). Inception-v4, inception-resnet and the impact of residual connections on learning, in Proceedings of the AAAI Conference on Artificial Intelligence, Vo. 31.

[B29] Szegedy C. Vanhoucke V. Ioffe S. Shlens J. Wojna Z. (2016). Rethinking the inception architecture for computer vision, in Proceedings of the IEEE Conference on Computer Vision and Pattern Recognition (Las Vegas, NV: IEEE), 2818–2826.

[B30] Szegedy C. Zaremba W. Sutskever I. Bruna J. Erhan D. Goodfellow I.. (2013). Intriguing properties of neural networks. arXiv [Preprint] arXiv:1312.6199.

[B31] Tramér F. Kurakin A. Papernot N. Goodfellow I. Boneh D. McDaniel P. (2017). Ensemble adversarial training: attacks and defenses. arXiv [Preprint] arXiv:1705.07204.

[B32] Vaswani A. Shazeer N. Parmar N. Uszkoreit J. Jones L. Gomez A. N.. (2017). Attention is all you need. In Advances in neural information processing systems, pages 5998–6008.

[B33] Wang X. He K. (2021). Enhancing the transferability of adversarial attacks through variance tuning, in Proceedings of the IEEE/CVF Conference on Computer Vision and Pattern Recognition (IEEE), 1924–1933.

[B34] Wang X. He X. Wang J. He K. (2021a). Admix: enhancing the transferability of adversarial attacks. arXiv [Preprint] arXiv: 2102.00436. 10.1109/CVPR46437.2021.00196

[B35] Wang Z. Guo H. Zhang Z. Liu W. Qin Z. Ren K. (2021b). Feature importance-aware transferable adversarial attacks. arXiv [Preprint] arXiv: 2107.14185.

[B36] Wu D. Wang Y. Xia S.-T. Bailey J. Ma X. (2020a). Skip connections matter: on the transferability of adversarial examples generated with resnets. arXiv [Preprint] arXiv: 2002.05990.

[B37] Wu L. Zhu Z. Tai C. others (2018). Understanding and enhancing the transferability of adversarial examples. arXiv [Preprint] arXiv:1802.09707.

[B38] Wu W. Su Y. Chen X. Zhao S. King I. Lyu M. R.. (2020b). Boosting the transferability of adversarial samples via attention, in 2020 IEEE/CVF Conference on Computer Vision and Pattern Recognition (CVPR) (Seattle, WA: IEEE), 1158–1167.

[B39] Wu W. Su Y. Lyu M. R. King I. (2021). Improving the transferability of adversarial samples with adversarial transformations, in Proceedings of the IEEE/CVF Conference on Computer Vision and Pattern Recognition, 9024–9033.

[B40] Xie C. Wang J. Zhang Z. Ren Z. Yuille A. (2017). Mitigating adversarial effects through randomization. arXiv [Preprint] arXiv:1711.01991.

[B41] Xie C. Zhang Z. Zhou Y. Bai S. Wang J. Ren Z.. (2019). Improving transferability of adversarial examples with input diversity, in Proceedings of the IEEE/CVF Conference on Computer Vision and Pattern Recognition, 2730–2739.

[B42] Xie P. Wang L. Qin R. Qiao K. Shi S. Hu G.. (2021). Improving the transferability of adversarial examples with new iteration framework and input dropout. arXiv [Preprint] arXiv:2106.01617.

[B43] Zhong Z. Zheng L. Kang G. Li S. Yang Y. (2020). Random erasing data augmentation. Proc. AAAI Conf. Artif. Intell. 34, 13001–13008. 10.1609/aaai.v34i07.7000

[B44] Zou J. Pan Z. Qiu J. Liu X. Rui T. Li W. (2020). Improving the transferability of adversarial examples with resized-diverse-inputs, diversity-ensemble and region fitting, in Computer Vision – ECCV 2020, Vol. 12367, eds Vedaldi A. Bischof H. Brox T. Frahm J.-M. (Cham: Springer International Publishing), 563–579.

